# A Hypomagnetic Field Aggravates Bone Loss Induced by Hindlimb Unloading in Rat Femurs

**DOI:** 10.1371/journal.pone.0105604

**Published:** 2014-08-26

**Authors:** Bin Jia, Li Xie, Qi Zheng, Peng-fei Yang, Wei-ju Zhang, Chong Ding, Ai-rong Qian, Peng Shang

**Affiliations:** Key Laboratory for Space Bioscience and Biotechnology, Institute of Special Environmental Biophysics, School of Life Sciences, Northwestern Polytechnical University, Xi'an, China; Université Jean Monnet, France

## Abstract

A hypomagnetic field is an extremely weak magnetic field—it is considerably weaker than the geomagnetic field. In deep-space exploration missions, such as those involving extended stays on the moon and interplanetary travel, astronauts will experience abnormal space environments involving hypomagnetic fields and microgravity. It is known that microgravity in space causes bone loss, which results in decreased bone mineral density. However, it is unclear whether hypomagnetic fields affect the skeletal system. In the present study, we aimed to investigate the complex effects of a hypomagnetic field and microgravity on bone loss. To study the effects of hypomagnetic fields on the femoral characteristics of rats in simulated weightlessness, we established a rat model of hindlimb unloading that was exposed to a hypomagnetic field. We used a geomagnetic field-shielding chamber to generate a hypomagnetic field of <300 nT. The results show that hypomagnetic fields can exacerbate bone mineral density loss and alter femoral biomechanical characteristics in hindlimb-unloaded rats. The underlying mechanism might involve changes in biological rhythms and the concentrations of trace elements due to the hypomagnetic field, which would result in the generation of oxidative stress responses in the rat. Excessive levels of reactive oxygen species would stimulate osteoblasts to secrete receptor activator of nuclear factor-κB ligand and promote the maturation and activation of osteoclasts and thus eventually cause bone resorption.

## Introduction

The geomagnetic field (GMF, ∼50 µT) is a natural component of the environment, and plays an important role in the growth and evolution of living organisms[1]. In addition, the GMF is essential for life and protects the Earth against high-energy particles from cosmic and solar winds[2]. Moreover, in deep-space exploration missions, biological objects transported on long-term interplanetary missions or on the surface of the moon or Mars would experience extremely weak magnetic fields—i.e., hypomagnetic fields (HMFs)—because the galactic magnetic field is 0.1–2 nT, whereas the lunar magnetic field is approximately 1000 times weaker than the Earth's GMF[3]–[5]. However, the effects of an HMF on the functions of biological organisms are still insufficiently understood despite active study.

A few studies have clearly confirmed that HMFs greatly affect the functional state and even the morphology of organisms[1],[6]–[8]. An experiment in which rabbits passed through embryogenesis and grew to an age of 1 month in an HMF environment indicated the development of degenerative disturbances in the liver, myocardium, and gastrointestinal tract; structural and energy metabolism perturbations; marked inhibition of enzymatic systems; and a significantly higher mortality rate compared with the controls[9]. In a shielded 5-nT environment, the incidence of somatic defects increased in the developing larvae of Japanese newts (*Cynops pyrrhogaster*); in particular, bi-headedness, intestinal protrusion, spinal curvature, malformed eyes, and retarded or blocked development were observed, especially after 20 days of shielding[10]. A brief 2-hour exposure to an HMF (<200 nT) is sufficient to interfere with the development of *Xenopus* embryos in the cleavage stages[11]. Long-term GMF deprivation results in animals exhibiting various behavioral and mood disorders such as inactivation, depression, mania, and anxiety, suggesting that their central nervous systems have been affected[12]. Adult male rats exposed to an HMF for 3 months exhibited significantly decreased work capacity, endurance, and behavioral activity as well as significant increases in heart rate and conditioned reflex development time[13]. After they were housed in an ambient 20-nT field for 7 months, the concentrations of certain elements in the hair of laboratory rats, especially iron, manganese, copper, and chromium, were significantly altered[14]. In addition, BALB/c mice housed in a GMF-shielding room (<300 nT) exhibited altered blood leukocyte and platelet counts at different times during the 28-day breeding period[15].

Bone loss in space is one of the most important problems endangering the health of astronauts. Previous studies suggest that the health of the skeletal system is dependent on Earth's gravity. Under microgravity conditions in space, the loss of bone calcium in the skeletal system, especially from the weight-bearing bones, is unavoidable; furthermore, continuous bone loss in space may cause fractures and renal calculus in astronauts, which may affect both their health and the mission[16], [17].

HMFs are extremely weak static magnetic fields, and are far weaker than the GMF. However, their effects on the skeletal system are unclear and have not been reported. Nevertheless, several studies demonstrate that a static magnetic field that is stronger than the GMF can affect bone remodeling and the activities of bone-related cells. An average flux of 290 mT in a static magnetic field accelerates the osteogenic differentiation and mineralization of dental pulp cells[18]. Exposure of an intramedullary implant to a static magnetic field (22–26 mT) radiologically improves bone healing in the first 2 weeks, and the difference in the configuration of the magnetic poles also affects bone quality[19]. In rabbit tibiae, the use of sand-blasted large-grid acid-etched titanium implants with a neodymium magnet (15.34 mT) can trigger quicker early peri-implant bone formation as compared with implants without a magnet[20]. A small disc magnet with a maximum magnetic flux density of 180 mT that was implanted in the right side of the spinous process of the third lumbar vertebra reportedly increased the bone mineral density (BMD) of osteoporotic lumbar vertebrae in ovariectomized rats[21].

As mentioned above, many previous studies indicate that not only gravity but also static magnetic fields can affect the skeletal system, and may thus play pivotal roles in maintaining skeletal system health. In space environments, microgravity is one of the complex factors, along with HMF and radiation that should be carefully considered with regard to its effects on astronauts. For astronauts who have acclimatized to the GMF, an HMF is a novel environmental factor. It remains unclear whether the absence of the GMF or HMF plus microgravity condition influences the skeletal system health. In an effort to provide scientific support to the health care of astronauts on deep-space missions, we investigated the effects of an HMF and the complex effects of an HMF plus hindlimb unloading (HLU) on bone loss in rats as well as the role and mechanism of HMF on bone loss in microgravity.

## Materials and Methods

### GMF-shielding chamber

**Figure 1:**
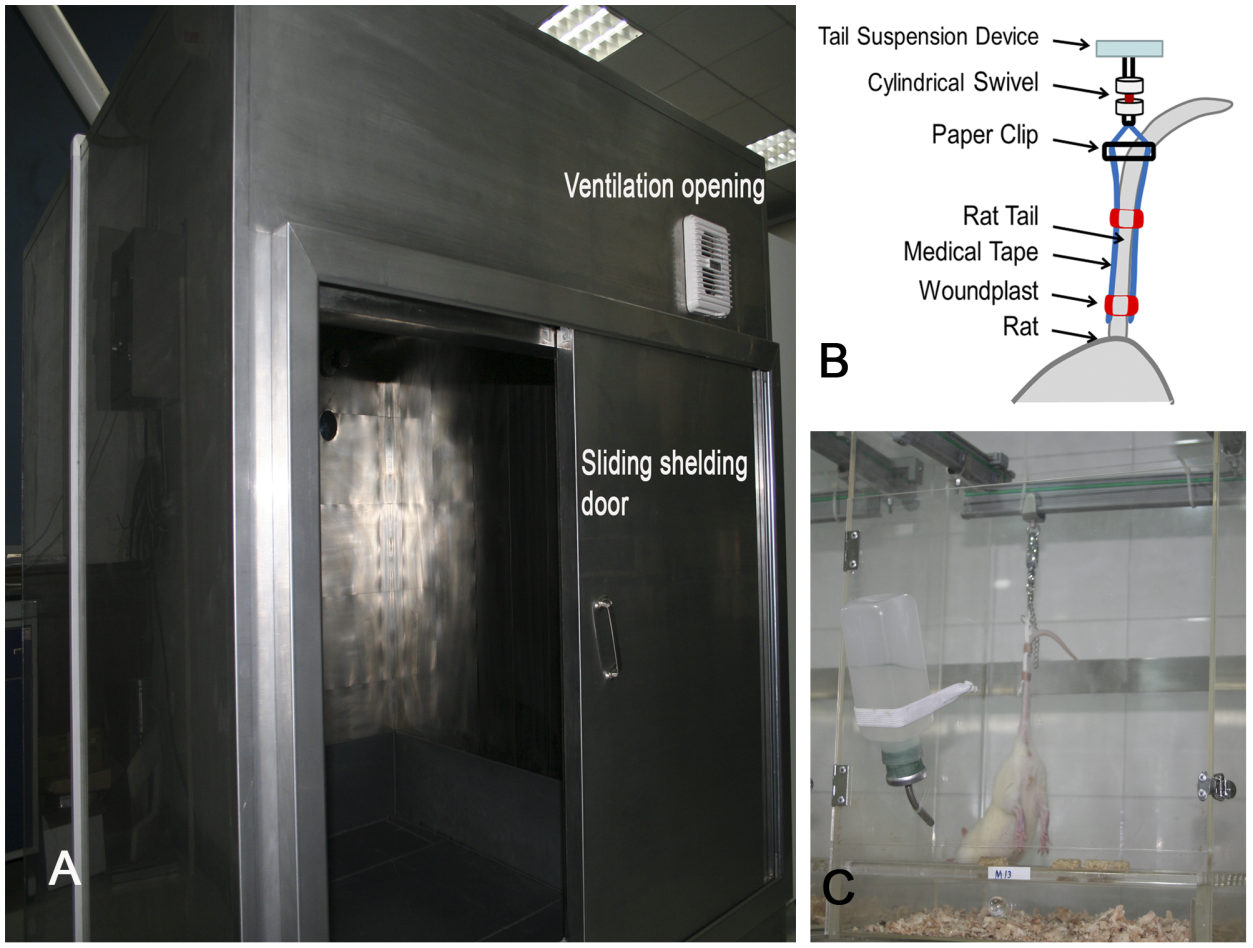
Hypomagnetic environment experimental device. (A) Geomagnetic field shielding chamber; (B) The schematic diagram of tail suspension; (C) Rats tail suspension cage.

To obtain a stable experimental HMF environment, a 1.8×1.6×1.5-m GMF-shielding chamber (NORINDAR International, Shijiazhuang, Hebei, China) was constructed (Figure 1A). The shielding chamber consisted of an aluminum alloy frame, several layers of highly permeable permalloy, silicon steel sheets, and pure iron sheets. The chamber can generate a hypomagnetic environment with an average magnetic field intensity of <300 nT. The mean magnetic field intensity in Xi'an, Shaanxi, China, is approximately 50 µT. A mandatory ventilation system was installed in the shielding chamber, with a ventilation frequency of 15 times per hour. Incandescent lights were used for illumination inside the shielding chamber (20 lm). The illumination switch was synchronized with a 12-h light/12-h dark cycle.

As a control, we constructed a 1.5×1.4×1.5-m wooden experimental box that has no shielding effect against the GMF but has the same illumination and ventilation conditions as the GMF shielding chamber.

### Experimental animals and tail-suspension animal model

A total of 60 male adult Sprague-Dawley rats weighing 260±10 g were provided by The Lab Animal Center of the Fourth Military Medical University, Xi'an, Shaanxi, China. The experiment was performed after the rats were allowed to acclimate for 3 days. During the experiment, each cage contained 1 HLU rat or 3 other rats, and feed and water were controlled. The entire experiment was performed in accordance with the recommendations of the local animal ethics association as well as the principles of laboratory animal welfare (Regulations for the Administration of Affairs Concerning Laboratory Animals in Shaanxi Province, China). The protocol was approved by the Lab Animal Ethics and Welfare Committee, Northwestern Polytechnical University (The Form of Animal Experimental Ethical Inspection, No. 10–17). Blood sample collection and execution were performed under sodium pentobarbital anesthesia, and all efforts were made to minimize suffering.

NASA's rat model involving tail suspension, HLU, and −30° downward head tilting was used [22]–[24] with some minor improvements to the fixation of the rat tails[25] (Figure 1C). Briefly, the strip-type medical adhesive tape pasted on both sides of the rat's tail was fixed by 2 flexible Band-Aid brand adhesive bandages (Johnson & Son, Racine, WI, USA), forming a ring around the tail. The part of the Band-Aid that touched the skin of the rat tail's was the nonsticky layer (Figure 1B). This improved fixation method can effectively avoid lesions on the rats' tails caused by improper tape fixation. Because the Band-Aid is made of elastic materials, the frequency of replacing the adhesive tape can be reduced during the 28-day experiment. Tail-suspended rats had access to food and water ad libitum. During the experiment, a few rats had learned to climb to the top of the cage using the cord used for tail suspension or by leaning their hind limbs against the cage wall, which led to model failure; such rats, accounting for 17.2% of animals that underwent tail suspension, were excluded from the analysis.

### Experimental design

Experimental animals were randomly divided into 5 groups: (1) the baseline group (BL), in which rats were executed to get basal data on day 0 of the experiment. (2) the control group, in which rats were kept inside the wooden experimental box with the normal GMF environment; (3) the HLU group, in which rats received tail suspension, HLU, and −30° downward head tilting, and were kept inside the wooden experimental box; (4) the HMF group, in which rats were kept inside the GMF-shielding chamber; and (5) the HMF+HLU group, in which HLU rats were kept inside the GMF-shielding chamber.

The experiment lasted for 4 weeks. On day 0, 28 of the experiment, the rats of BL group and other groups were sent for a BMD scan and serum sample collection from the inferior vena cava under anesthesia respectively. The rats were subsequently killed by cervical vertebra luxation under anesthesia, and their bilateral femurs were separated from the soft tissues. For biomechanical analysis, the left femur was immediately bisected and fixed with 4% paraformaldehyde. Decalcification treatment was also performed before the femur was sent for immunohistochemical analysis. The right femur was used to obtain micro-CT scan data.

### BMD measurement

Dual-energy x-ray absorptiometry (DEXA) (Lunar Prodigy; GE Medical Systems, Madison, WI, USA) in the small-animal mode was used to measure the BMD of femurs in vivo. On day 0, 28 of the experiment, BL group and all experimental animals were anesthetized with an intraperitoneal injection of 3% pentobarbital sodium (1 mL/kg) and placed in the prone position with the lower limbs naturally extended on the absorptiometry machine for BMD measurement. The scan results were analyzed with enCORE software version 10.50 (GE Medical Systems) for assessing the BMD and bone mineral content (BMC) of bilateral femurs.

### Micro-CT femur 3D reconstruction and analysis

An eXplore Locus SP micro-CT (GE Medical Systems) was used for femur 3D reconstruction and analysis. The scan parameters were as follows: scan resolution, 14 µm; rotation angle, 360°; rotation angle increment, 0.4°; voltage, 80 kV; current, 80 µA; exposure time, 3000 ms. After the scan was completed, MicroView™ (version 2.1.2) was used for 3D reconstruction. Analysis was performed with MicroView™ Advance Bone Analysis(ABA)-specific bone analysis software. A 2.019-mm-thick trabecular bone chip under the epiphyseal plate in the lower end of the femur was selected as the region of interest (ROI) for 3D reconstruction and measurements. The following parameters were measured: BMC, BMD, tissue mineral content (TMC), tissue mineral density (TMD), structure model index (SMI)[26], bone volume fraction (BVF, i.e., the ratio of bone volume to tissue volume [BV/TV]), bone surface to bone volume (BS/BV), trabecular thickness (Tb.Th), trabecular separation (Tb.Sp), degree of anisotropy, Euler number[27], and connectivity density (1/mm3)[28]. A 1.512-mm-thick cortical bone chip in the middle of the femur was selected as the ROI for 3D reconstruction and measurements of BMC, BMD, mean thickness, inner perimeter, outer perimeter, marrow area, and cortical area.

### Mechanical properties

On day 28 of the experiment, the rats were anesthetized and killed by cervical dislocation, and the soft tissues of both hind limbs and the bilateral femurs were removed. The mechanical properties of the femurs were tested using the conventional 3-point bending test. A universal testing machine (Instron, Canton, MA, USA) was used to support the platform. The span between 2 load-supporting points was set at 20 mm. Femurs from each experimental group were placed on the supporting platform in the same orientation, and load was added evenly at the midpoint of the femur at 2 mm/min until the femur fractured. A load-deformation curve was simultaneously obtained during loading, through which the mechanical properties of the femur were determined, including ultimate force, toughness factor, and elastic modulus.

### Biochemical analysis

The abdominal wall was cut open under anesthesia to expose the abdominal aortic vein. A syringe was then used to directly collect blood from the abdominal aortic vein. After the blood sample was collected, the animal was killed by cervical dislocation. The collected blood sample was stored at 4°C for 1 h and then centrifuged at 1500 rpm for 10 min. Serum was collected by suction and stored in a fridge at −70°C. The serum concentrations of bone alkaline phosphatase (bALP), deoxypyridinoline (DPD), and glucocorticoids (GCs) were determined using an enzyme-linked immunoassay detection kit (Beijing Chenglin Biotechnology Co. Ltd.(Beijing, China); primary antibodies were obtained from Abcam(Cambridge, UK)).

Trace elements in serum were detected by an atomic absorption spectrophotometer (ZEEnit700P; Analytik Jena AG, Jena, Germany). Serum manganese was detected using the graphite furnace atomic absorption spectroscopy method, whereas other elements including iron, copper, zinc, calcium, and magnesium, were detected using the flame method.

### Immunohistochemistry

After biomechanical tests, fresh femurs were split at the center line and fixed in 4% paraformaldehyde for 24 hours. The paraformaldehyde solution was then discarded, and decalcification solution (20% EDTA) was added and subsequently changed every 4 days. Decalcification was performed continuously for 28 days. The decalcified femurs were dehydrated and embedded in paraffin to prepare paraffin sections. After dewaxing, the streptavidin-biotin complex method was used to detect the expression of receptor activator of nuclear factor-κB ligand (RANKL) in bone tissue, by using its antibody (anti-RANKL; Abcam). A semiquantitative analysis was conducted for determining the percentage of RANKL-positive cells and the intensity of positivity, which was classified into 4 degrees (0, negative; 1, slightly positive; 2, distinctly positive; and 3, strongly positive). Moreover, the intensity of RANKL positivity was quantified as the H-score, where H-score  =  ∑(i + 1) × Pi, in which i is the degree of positivity as classified above (from 0–3) and Pi is the percentage of the positive cells which fluctuates from 0% to 100%. Five vision fields (× 400) were randomly selected on each section, and RANKL-positive cells were counted in accordance with the above method.

### Statistics

All statistical analyses were performed by using the GraphPad Prism for Windows statistical software (version 5, GraphPad Software, Inc., La Jolla, CA, USA). The differences between the BL group and the other experimental groups were revealed using an ordinary one-way ANOVA. Two-way analysis of variance was applied to test the variation trends and differences between the experimental groups. The results are expressed as mean ± standard deviation. For all statistical tests, a *P* value of <0.05 was considered to be statistically significant.

## Results

### Effects of HMF and HMF+HLU on the body weight of rats

**Figure 2:**
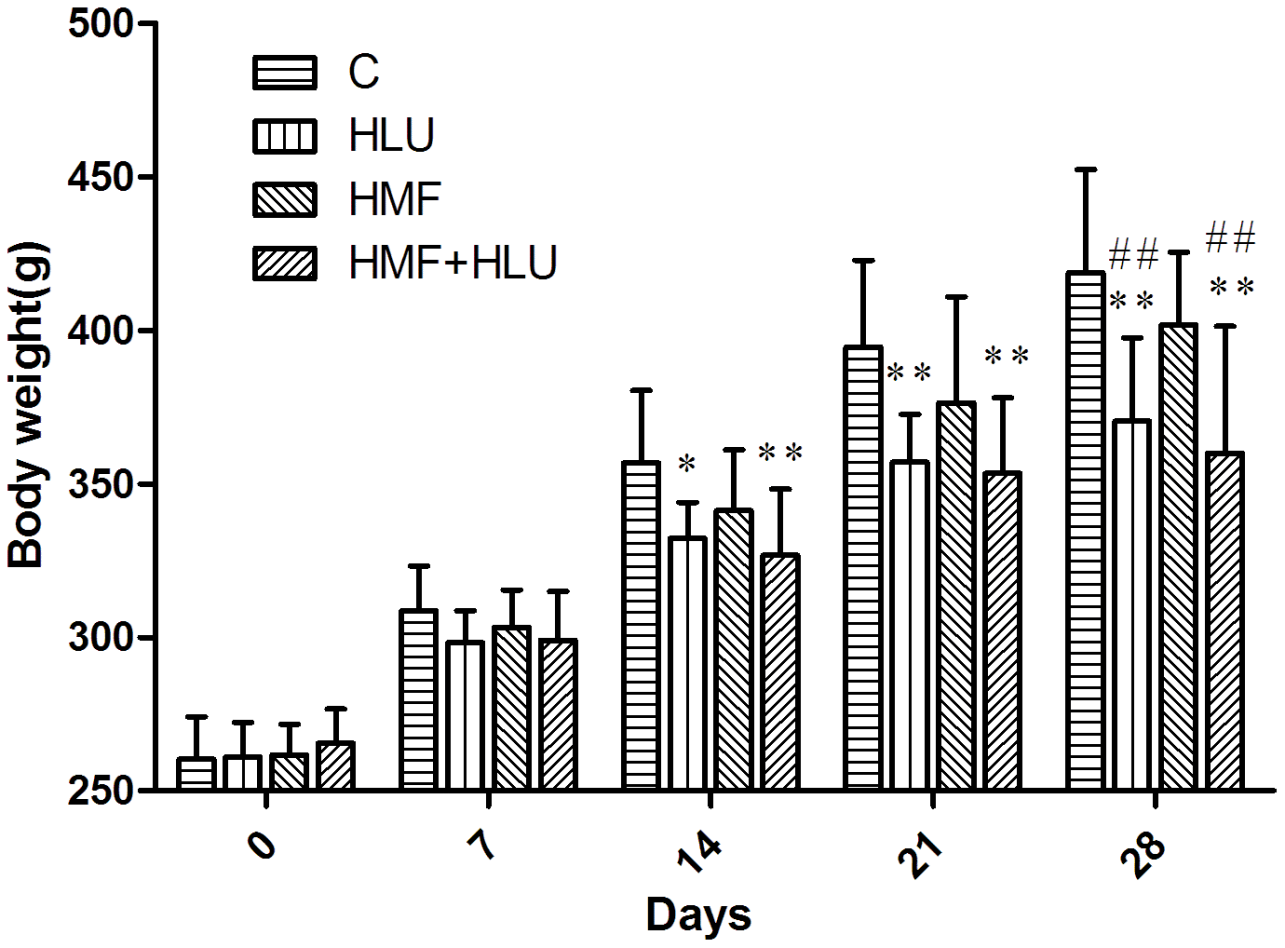
Changes in bodyweight from the original weight of rats in different groups. Rats in all 4 groups were weighed every 7d. C: Rats were raised in a wooden box with a normal GMF for 28 days; HLU: Rats were suspended, unloaded with −30° downward head tilting, and raised in a wooden box; HMF: Rats were raised normally in a GMF-shielded room; HMF+HLU: HLU rats were raised in a GMF-shielded room. **P<0.01 *vs* C, *P<0.05 *vs.* C, ^##^ *P<0.01 vs.* HMF.

The differences in body weights between the experimental groups except the BL group are shown in Figure 2. During the experimental period, the body weight in all the rats increased. The body weights of the rats in the HLU and HMF+HLU groups were significantly decreased compared with those of the rats in the control group from days 14 to 28 (*P<*0.05 in the HLU group at 14 days, *P<*0.01 in the HLU and HMF+HLU group at 21 and 28 days.). At day 28, compared with the HMF group, the body weights of the rats in the HLU and HMF+HLU groups were significantly decreased (*P<*0.01).

### Effects of HMF and HMF+HLU on the mechanical properties of the rat femur

**Figure 3:**
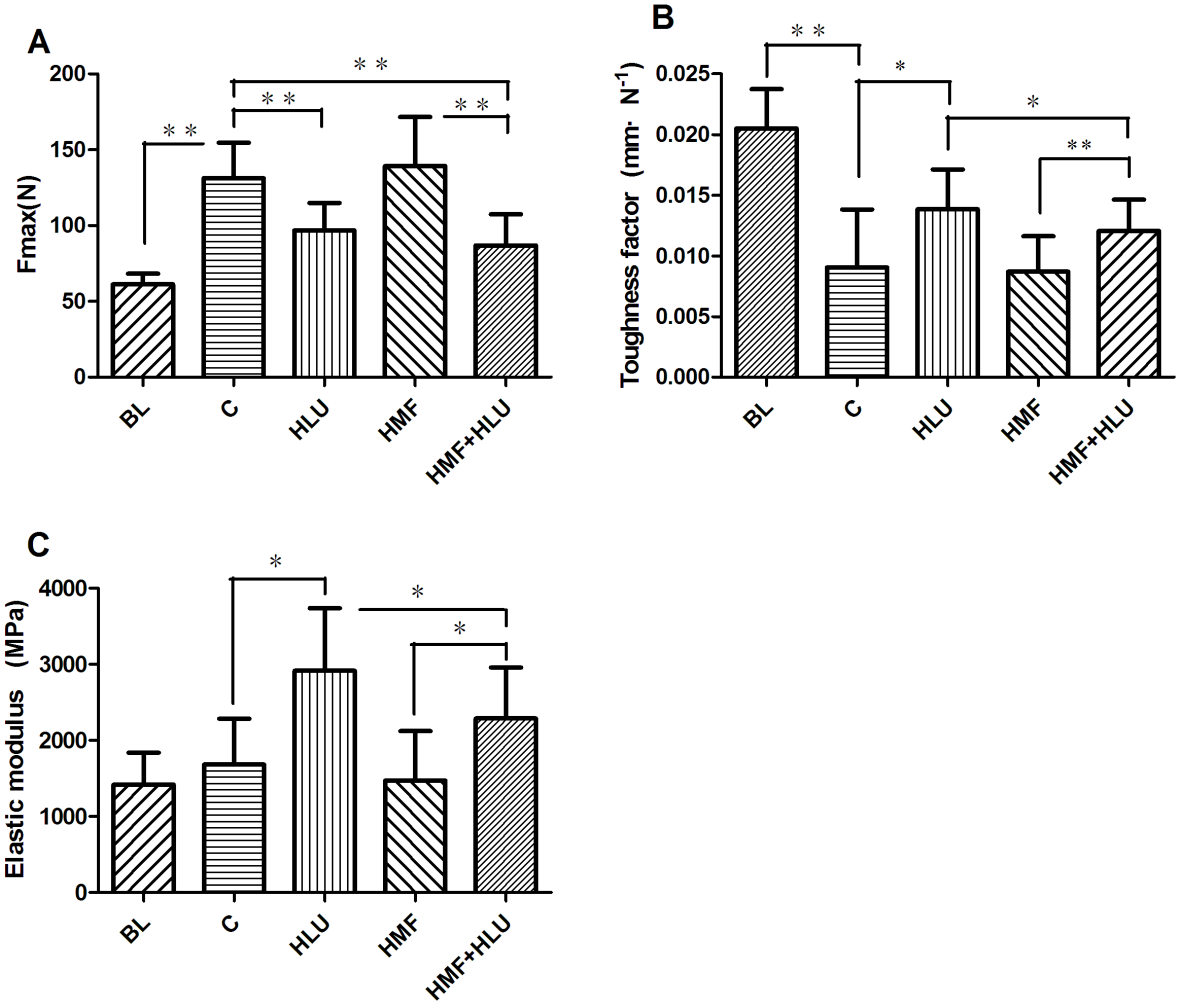
Mechanical testing results. The ultimate force (A), toughness factor (B), and elastic modulus (C) were obtained by analyzing the load–deformation curve. BL:The baseline group. Rats were executed to get basal data on day 0 of the experiment. C: Rats were raised in a wooden box with a normal GMF for 28 days; HLU: Rats were suspended, unloaded with −30° downward head tilting, and raised in a wooden box; HMF: Rats were raised normally in a GMF-shielded room; HMF+HLU: HLU rats were raised in a GMF-shielded room.**P<0.01,*P<0.05.

The mechanical properties of the femur in the 3-point bending test are shown in Figure 3. The ultimate load (Fmax) in the control, HLU, HMF and HMF+HLU groups were significantly increased, compared with BL group. The HLU and HMF+HLU groups had significantly lower Fmax than the control and HMF groups (*P*<0.01); however, according to two-way ANOVA, the interaction between the two factors was no statistically significant. Compared with BL group, the toughness factor of all experiment groups were decreased obviously. For the toughness factor, there was a significant effect for HLU (*P*<0.05) and HMF (*P*<0.05). Likewise, the interaction HLU*HMF was significant (*P*<0.05). The post-hoc test revealed that the toughness factor, in the HMF+HLU group, was significantly greater than that of the HMF group, but significantly lower than that of the HLU group (*P*<0.05). Moreover, the elastic modulus of the HMF+HLU group was significantly greater than that of the HMF group (*P*<0.05), but significantly lower than that of the HLU group (*P*<0.05). The elastic modulus of the BL group was similar to the control and HMF group was lower than that of the HLU and HMF+HLU groups.

### Effects of HMF and HMF+HLU on femoral BMD and BMC

**Figure 4:**
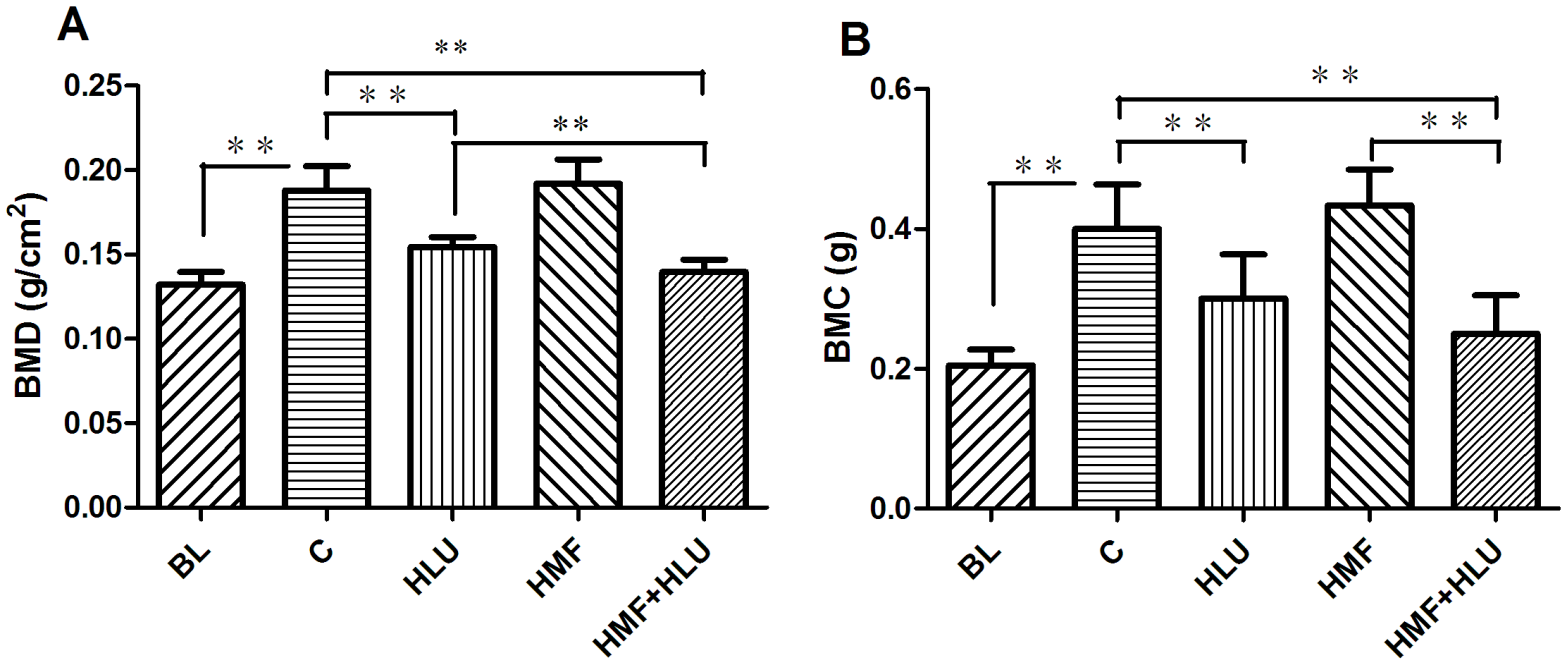
Changes in femoral BMD and BMC measured by DEXA. BL: The baseline group. Rats were executed to get basal data on day 0 of the experiment. C: Rats were raised in a wooden box with a normal GMF for 28 days; HLU: Rats were suspended, unloaded with −30° downward head tilting, and raised in a wooden box; HMF: Rats were raised normally in a GMF-shielded room; HMF+HLU: HLU rats were raised in a GMF-shielded room. BMD: bone mineral density; BMC: bone mineral content; **P<0.01.

DEXA analysis through two-way ANOVA showed that there was a significant effect for HLU (*P*<0.01) and HMF (*P*<0.01) in the femoral BMD and the interaction HLU*HMF was significant (*P*<0.05) in the femoral BMD. For the femoral BMC, the interaction HLU*HMF was no significant. Further analysis, the femoral BMD and BMC of the HLU and HMF+HLU groups were significantly less than those of the control and HMF groups (all *P*<0.01). The femoral BMD was lower in the HMF+HLU group than in the HLU group (*P*<0.01), whereas the BMC of the femurs was similar between groups. The differences in femoral BMD and BMC were not significant between the HMF and control groups (Figure 4). BMD and BMC of all experimental groups were higher than the BL groups except the BMD of the HMF+HLU group.

### Effects of HMF and HMF+HLU on femoral trabecular and cortical bone

**Figure 5:**
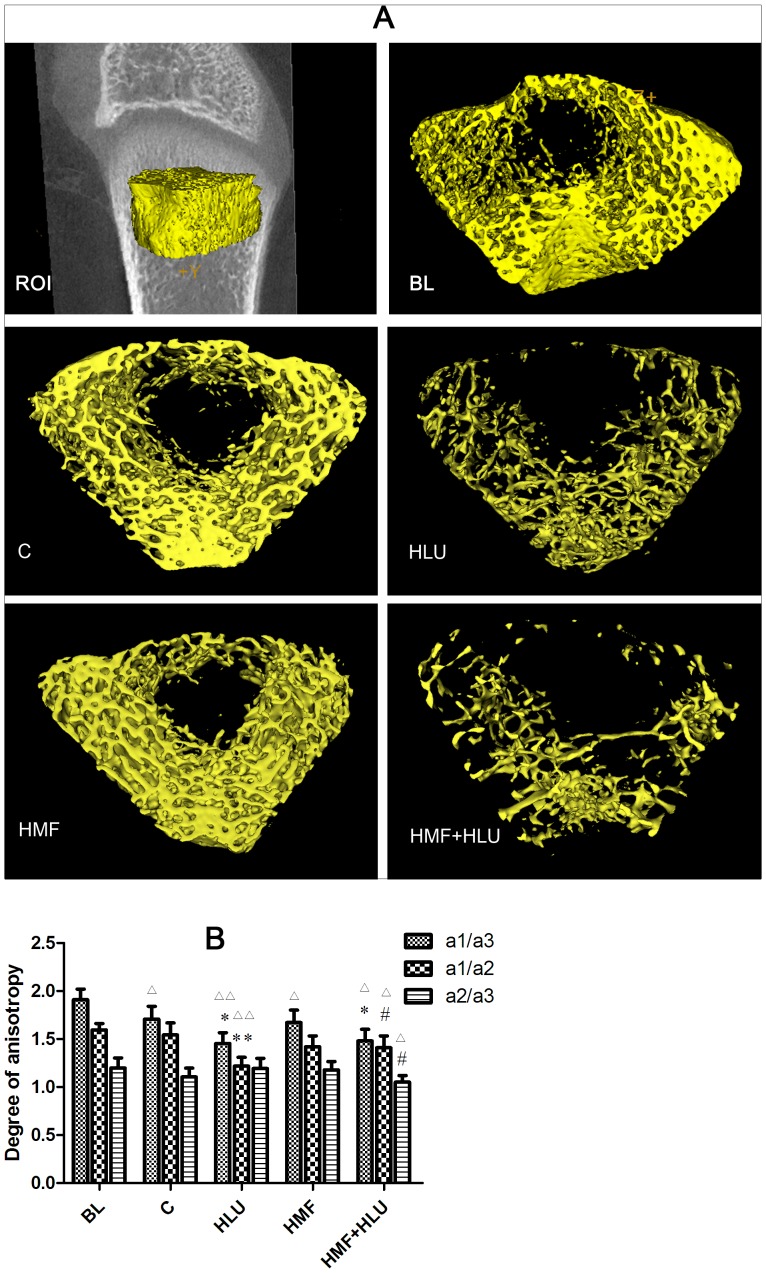
Effects of HMF and HMF+HLU on the femur trabecular bone. ROI: A 2.019-mm-thick trabecular bone chip under the epiphyseal plate in the lower end of the femur was selected as the region of interest (ROI). BL: The baseline group. Rats were executed to get basal data on day 0 of the experiment. C: Rats were raised in a wooden box with a normal GMF for 28 days; HLU: Rats were suspended, unloaded with −30° downward head tilting, and raised in a wooden box; HMF: Rats were raised normally in a GMF-shielded room; HMF+HLU: HLU rats were raised in a GMF-shielded room. **(A)** Three-dimensional trabecular bone architecture of an ROI located under the epiphysis plate of the femur. **(B)** The degree of anisotropy is a measure of the extent of the orientation of the substructures in an ROI of trabecular bone, which represents the directivity and symmetry of the trabecular structure; a1/a3, a1/a2, and a2/a3 are the ratios of the long to the short diameters on 3 mutually perpendicular plane ellipses inside the ellipsoid. **P<0.01 vs. C, *P<0.05 vs. C, ^#^ *P<*0.05 vs. HLU, ^△△^ *P<*0.01 vs. BL, ^△^ *P<*0.05 vs. BL.

The three-dimensional (3D) reconstruction of the 2.019-mm-thick trabecular bone ROI at the epiphyseal end of the femur (Figure 5A) by MicroView™ version 2.1.2 revealed significant differences in the ROI of the femurs of HLU rats in a normal GMF environment compared with that in the control group, with both the thickness and number of bone trabeculae significantly reduced. The femoral ROI of the HMF rats showed no significant differences compared with that of the control group, although the femoral ROI of HMF+HLU rats showed significant differences compared with that of the control, HMF, and HLU groups; moreover, the HMF+HLU group showed more significant bone trabecular changes than the HLU group. Compared with the BL group, only the BVF in the control and HMF groups, the BMC in the HMF group were no significant differences (Table 1).

**Table 1:** Microarchitectural quantitative analysis results of an ROI in the femur trabecular bone.

	BL	C	HLU	HMF	HMF+HLU
BMD (mg/cm^3^)	274.97±25.46	340.82±32.72^△△^	168.56±13.42^**△△^	340.53±37.17^△△^	152.50±10.62^**##△△^
TMD (mg/cm^3^)	418.65±6.86	543.40±68.53^△△^	504.61±54.26^△△^	553.33±48.44^△△^	524.45±56.64^△△^
BMC (mg)	8.34±1.19	9.876±1.214^△^	4.004±0.506^**△△^	8.896±1.014	3.252±0.407^**##△△^
TMC (mg)	4.93±1.07	6.254±0.652^△△^	1.444±0.125^**△△^	5.704±0.625^△^	0.942±0.124^**##△△^
SMI	0.7612±0.428	0.106±0.008^△△^	2.246±0.165^**△△^	−0.175±0.013^**△△^	2.106±0.163^**△△^
BVF	0.388±0.07	0.392±0.026	0.121±0.007^**△△^	0.395±0.032	0.084±0.005^**##△△^
EUN	−4068.3±618	−1804.5±127.6^△△^	−608.5±53.32^**△△^	−1684.3±176.4^△△^	−341.8±36.56^**##△△^
COD (1/mm3)	138.45±18.12	64.76±5.42^△△^	26.64±2.14^**△△^	67.06±7.11^△△^	16.05±1.31^**##△△^

BMD, bone mineral density (mg/cm^3^), refers to the total BMD of the ROI; BMC, bone mineral content (mg), refers to the total BMC of the ROI, including the ossature and soft tissue; TMC, tissue mineral content (mg), refers to the mineral content of the ossature of the ROI; TMD, tissue mineral density (mg), refers to the mineral density of the ossature of the ROI; SMI, structure model index, is a method for determining the plate- or rod-like geometry of trabecular structures; BVF, bone volume fraction, refers to the ratio of bone volume to tissue volume; EUN,Euler number indicates the decrease in osteoporosis; COD, connectivity density shows the number of connections in the trabecular networks, which is decreased in osteoporosis. **P<0.01vs. C, ##*P<*0.01vs.HLU, ^△△^ *P<*0.01vs.BL, ^△^ *P<*0.05vs.BL.

Two-way ANOVA revealed a significant effect for HLU (*P*<0.01) and HMF (*P*<0.01) in the femoral BMD, BMC, TMC, BVF, EUN and COD. Likewise, the interaction HLU*HMF was significant (*P*<0.01). The 3D BMD and BMC values were significantly lower in the HMF+HLU group than in the control, HLU, and HMF groups (*P*<0.01); however, no significant differences in these values were observed between the HMF and control groups. The 3D TMC was significantly lower in the HMF+HLU group than in the control, HLU, and HMF groups (*P*<0.01); however, no significant difference was observed in these values between the HMF and control groups. There were no significant differences between groups with respect to TMD (Table 1).

The SMI of the HMF+HLU group differed significantly from that of the control and HMF groups (*P*<0.01); however, there was also a significant difference in this value between the HMF and control groups (*P*<0.01). The BVF of the HMF+HLU group was significantly different from that of the control and HMF groups (*P*<0.01). The absolute value of the Euler number and connectivity density were significantly lower in the HMF+HLU group than in the control, HLU, and HMF groups (*P*<0.01); however, no significant differences were observed in these values between the HMF and control groups (Table 1).

With regard to the degree of anisotropy, the a1/a3 plane value of the HMF+HLU and HLU groups was significantly lower than that of the control group and BL respectively (*P*<0.05, *P*<0.01), and the a1/a2 plane value of the HLU group was significantly lower than that of the control group and BL (*P*<0.01). Moreover, the degrees of anisotropy of the a1/a2 and a2/a3 planes of the HMF+HLU group were not significantly different from those of the control group and BL, and were significantly lower than those of the HLU group (*P*<0.01) and BL (*P*<0.05) (Figure 5B).

The axial values of BV/TV at the x, y, and z axes as well as the mean values exhibited consistent changes. Two-way ANOVA revealed a significant effect for HLU (*P*<0.01) and HMF (*P*<0.01) in BV/TV, Tb.N and Tb.Sp. The interaction HLU*HMF was significant (*P*<0.01). The BV/TV of the HMF+HLU group differed significantly from those of the HLU and control groups (*P*<0.01). The axial values of BS/BV at the x, y, and z axes as well as the mean values were significantly higher in the HMF+HLU and HLU groups than in the control group (*P*<0.01); however, no significant differences in these values were observed between the HMF+HLU and HLU groups. The Tb.Th of the HMF+HLU and HLU groups was significantly lower than that of the control group (*P*<0.01); however, no significant differences in this value were observed between the HMF+HLU and HLU groups. The trabecular number of the HMF+HLU and HLU groups were significantly lower than that of the control group (*P*<0.01); however, the trabecular number of the HMF+HLU group was significantly higher than that of the HLU group (*P*<0.01). Furthermore, the Tb.Sp of the HMF+HLU and HLU groups was significantly higher than that of the control group (*P*<0.01); however, the Tb.Sp was significantly higher in the HMF+HLU group than in the HLU group (*P*<0.01). Compared with BL, significant differences in BS/BV, Tb.Th, Tb.N and Tb.Sp of other groups were observed (*P*<0.01 or *P*<0.05); however, no significant differences in BV/TV of control and HMF groups were observed (Table 2).

**Table 2:** The axial values of microarchitectural quantitative analysis of an ROI in the femurtrabecular bone.

		BV/TV(%)	BS/BV(1/mm)	Tb.Th(mm)	Tb.N(1/mm)	Tb.Sp(mm)
BL	Axis x	0.39±0.07	34.3±3.57	0.059±0.006	6.56±0.57	0.095±0.018
	Axis y	0.39±0.07	34.5±3.95	0.059±0.007	6.56±0.43	0.094±0.016
	Axis z	0.39±0.07	19.4±2.17	0.104±0.012	3.70±0.32	0.168±0.031
	Avg	0.39±0.07	29.4±3.20	0.069±0.008	5.61±0.44	0.111±0.020
C	Axis x	0.40±0.03	23.04±2.06^△△^	0.087±0.008^△△^	4.57±0.38^△△^	0.132±0.010^△^
	Axis y	0.40±0.03	23.96±2.57^△△^	0.084±0.007^△△^	4.75±0.41^△△^	0.127±0.009^△^
	Axis z	0.40±0.03	12.77±1.03^△△^	0.157±0.017^△△^	2.53±0.22^△△^	0.238±0.022^△^
	Avg	0.40±0.03	19.92±1.89^△△^	0.100±0.008^△△^	3.95±0.31^△△^	0.153±0.014^△^
HLU	Axis x	0.12±0.01^**△△^	41.79±3.98^**△△^	0.048±0.004^**△△^	2.51±0.20^**△△^	0.350±0.030^**△△^
	Axis y	0.12±0.01^**△△^	44.04±3.58^**△△^	0.045±0.004^**△^	2.65±0.24^**△△^	0.332±0.030^**△△^
	Axis z	0.12±0.01^**△△^	26.57±2.71^**△△^	0.075±0.007^**△△^	1.60±0.13^**△△^	0.551±0.040^**△△^
	Avg	0.12±0.01^**△△^	37.47±3.42^**△△^	0.053±0.004^**△^	2.25±0.22^**△△^	0.391±0.033^**△△^
HMF	Axis x	0.39±0.04	25.40±2.22^△△^	0.079±0.005^△△^	5.00±0.45^△△^	0.121±0.009
	Axis y	0.39±0.04	22.73±2.54^△△^	0.088±0.007^△△^	4.48±0.39^△△^	0.135±0.012^△^
	Axis z	0.39±0.04	13.59±1.26^△△^	0.147±0.011^△△^	2.68±0.20^△△^	0.227±0.019^△^
	Avg	0.39±0.04	20.58±2.01^△△^	0.097±0.008^△△^	4.05±0.35^△△^	0.150±0.014^△^
HMF+HLU	Axis x	0.09±0.01^**##△△^	41.85±3.76^**△△^	0.048±0.004^**△△^	1.97±0.16^**##△△^	0.461±0.041^**##△△^
	Axis y	0.09±0.01^**##△△^	41.09±3.71^**△△^	0.049±0.004^**△△^	1.93±0.13^**##△△^	0.469±0.042^**##△△^
	Axis z	0.09±0.01^**##△△^	25.72±2.01^**△△^	0.078±0.007^**△△^	1.21±0.10^**##△△^	0.749±0.054^**##△△^
	Avg	0.09±0.01^**##△△^	36.22±3.16^**△△^	0.055±0.005^**△△^	1.70±0.13^**##△△^	0.532±0.046^**##△△^

BV/TV, bone volume to tissue volume (%); BS/BV, bone surface to bone volume (1/mm); Tb.Th, trabecular thickness (mm); Tb.N, trabecular number (1/mm); Tb.Sp, trabecular separation (mm); The x, y, and z axes express the quantities of the 3D structure; Avg, average quantities of the vector sum of the x, y, and z axes.*P<0.05 vs. C, **P<0.01 vs. C, #*P<*0.05 vs. HLU, ##*P*<0.01 vs. HLU, ^△△^ *P*<0.01 vs. BL, ^△^ *P*<0.05 vs. BL.

The 3D reconstruction of the 1.5012-mm-thick cortical bone ROI at the middle of the femur yielded the following findings (Table 3): compared with the control group, the BMC, mean thickness, and cortical area in the HLU and HMF+HLU groups were significantly reduced; the HMF group showed no significant differences compared with the control group; and the HMF+HLU group showed significant differences compared with the HLU groups in terms of the BMC and cortical area. Compared with the BL group, the BMC, mean thickness, inner Perimeter and cortical area in the HLU group,the BMD, BMC and mean thickness in HMF+HLU group, the mean thickness in HMF group were no significant changes. Certainly,the work preceding the above-mentioned analysis revealed that, only in BMC (*P*<0.01) and Cortical Area (*P*<0.05), the interaction HLU*HMF was significant.

**Table 3:** Microarchitectural quantitative analysis results of an ROI in the femur cortical bone.

	BL	C	HLU	HMF	HMF+HLU
BMD (mg/cm^3^)	834.91±29.31	958.36±65.30^△△^	927.56±57.54^△^	946.53±68.59^△△^	865.50±36.87
BMC (mg)	0.116±0.001	0.172±0.03^△△^	0.121±0.03^**^	0.168±0.02^△△^	0.090±0.01^**##^
Mean Thickness(mm)	0.474±0.040	0.543±0.069^△^	0.394±0.09^**^	0.458±0.116	0.368±0.102^**^
Inner Perimeter(mm)	9.374±0.743	11.83±1.58^△△^	10.18±1.17^*^	11.42±2.00^△^	11.75±1.16^△△^
Outer Perimeter(mm)	12.319±0.548	14.94±1.54^△△^	13.5±1.26^*△^	14.82±1.89^△△^	14.32±1.68^△△^
Marrow Area(mm^2^)	6.36±0.94	9.72±1.23^△△^	8.65±1.10^*△△^	9.64±1.31^△△^	9.5±1.57^△△^
Cortical Area(mm^2^)	4.85±0.31	6.79±0.60^△△^	4.57±0.35^**^	6.57±0.55^△△^	3.74±0.43^**#△^

BMD, bone mineral density (mg/cm^3^), refers to the total BMD of the ROI; BMC, bone mineral content (mg), refers to the total BMC of the ROI, including the ossature and soft tissue; Mean Thickness, average thickness of cortical bone; Inner Perimeter, the inside perimeter of the cortical bone; Outer Perimeter, the outside perimeter of the cortical bone; Marrow Area, the area of the cortical bone inside; Cortical Area, the area of the cortical bone.*P*<*0.05 vs. C, **P<0.01vs. C, #*P<*0.05vs.HLU, ##*P<*0.01vs.HLU, ^△△^ *P<*0.01vs.HL, ^△^ *P<*0.05vs.HL.

### Effects of HMF and HMF+HLU on RANKL expression in the femur

**Figure 6:**
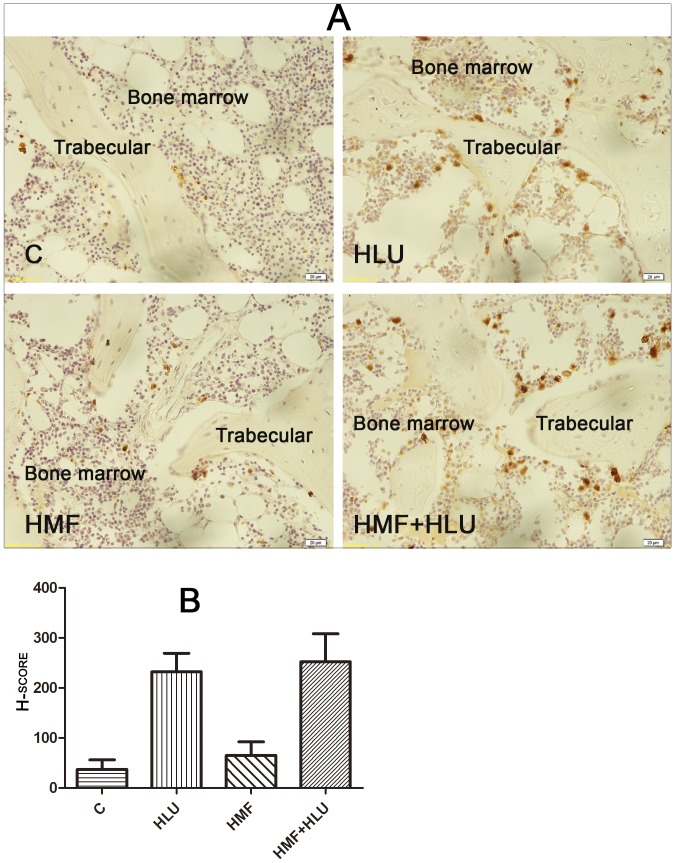
Effects of HMF and HMF+HLU on RANKL expression in femur. C: Rats were raised in a wooden box with a normal GMF for 28 days; HLU: Rats were suspended, unloaded with −30° downward head tilting, and raised in a wooden box; HMF: Rats were raised normally in a GMF-shielded room; HMF+HLU: HLU rats were raised in a GMF-shielded room. (A) Immunohistochemical staining of RANKL expression in femur. Brown cells are RANKL expression positive cells. (400×); (B) Semi-quantitative data figure of RANKL expression. HSCORE 100∼400: positive expression.

The expression of RANKL could be clearly observed in the femoral trabecular bone in the HLU and HMF+HLU groups, whereas no obvious expression was observed in the control or HMF group (Figure 6A). A semiquantitative analysis yielded a positive result in the HLU and HMF+HLU groups, with H-scores that were much higher than 100. The H-scores in the control and HMF groups were less than 100—a negative finding (Figure 6B).

### Effects of HMF and HMF+HLU on serum bALP, DPD and GC concentrations

**Figure 7:**
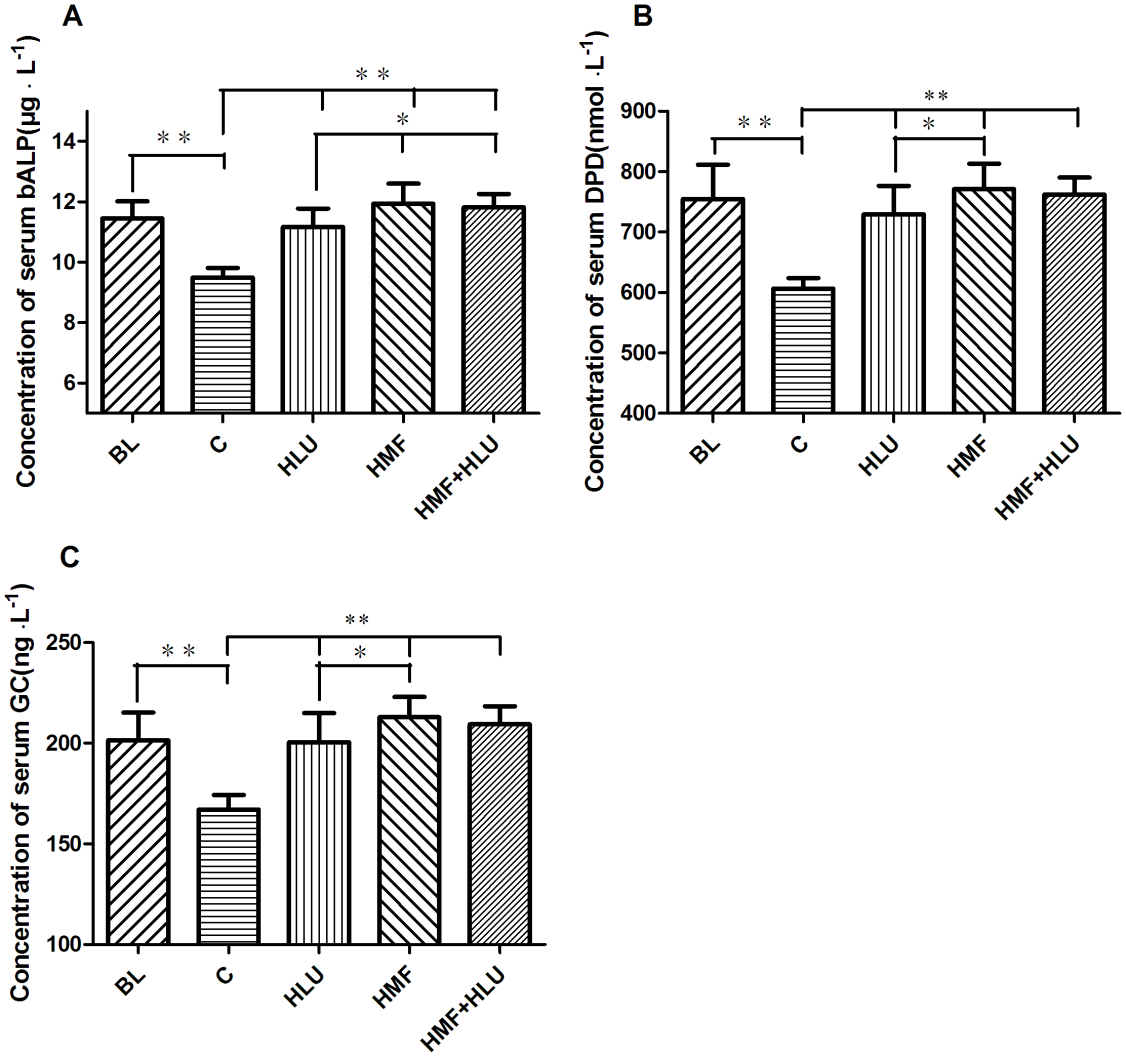
Effects of HMF and HMF+HLU on the concentrations of serum bALP, DPD and GCs. BL: The baseline group. Rats were executed to get basal data on day 0 of the experiment. C: Rats were raised in a wooden box with a normal GMF for 28 days; HLU: Rats were suspended, unloaded with −30° downward head tilting, and raised in a wooden box; HMF: Rats were raised normally in a GMF-shielded room; HMF+HLU: HLU rats were raised in a GMF-shielded room. Serum concentrations of (A) bALP (µg⋅L^−1^), (B)DPD (nmol⋅L^−1^), and (C) GCs (ng⋅L^−1^). **P<0.01.

The serum bALP, DPD, and GC concentrations in each group exhibited consistent changes (Figure 7). Two-way ANOVA revealed a significant effect for HLU (*P*<0.01) and HMF (*P*<0.01) in bALP, DPD, and GC concentrations. The interaction HLU*HMF was significant (*P*<0.01). These concentrations were significantly greater in the HLU, HMF, and HMF+HLU groups than in the control group (*P*<0.01). Furthermore, the HMF and HMF+HLU groups also exhibited significant differences in these values compared with the HLU group; in particular, bALP concentrations were significantly higher in the HMF and HMF+HLU groups than in the HLU group (*P*<0.05). Moreover, DPD and GC concentrations differed significantly between the HMF and HLU groups (*P*<0.05); furthermore, the HMF+HLU groups exhibited an increasing trend in these values compared with the HLU group, although the difference was not significant. Compared with BL group, the bALP, PDP and GC concentrations in control group were significantly decreased, and other groups were no significant changes.

### Effects of HMF and HMF+HLU on serum trace element concentrations

**Figure 8:**
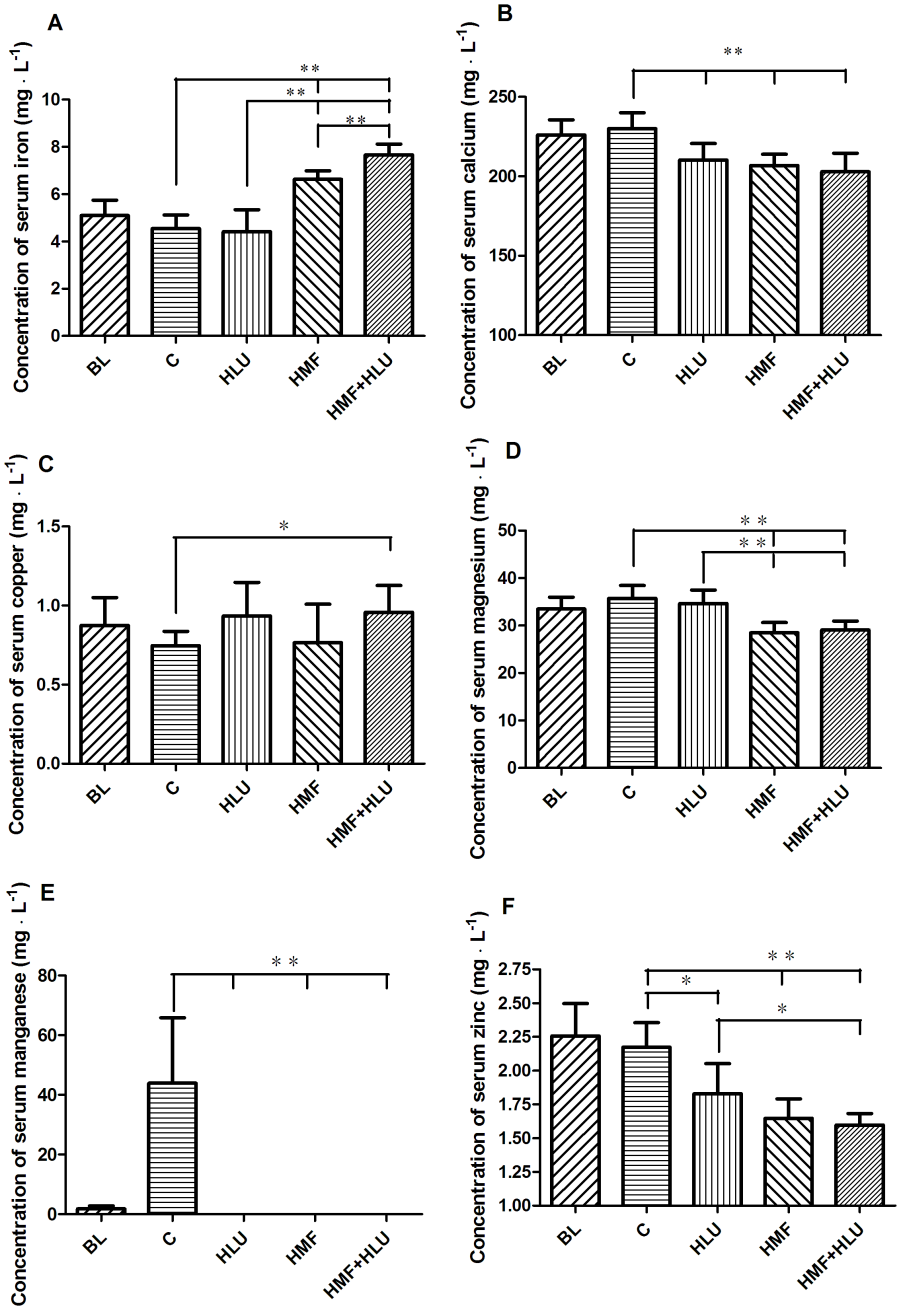
Effects of HMF and HMF+HLU on the concentrations of serum trace elements. BL: The baseline group. Rats were executed to get basal data on day 0 of the experiment. C: Rats were raised in a wooden box with a normal GMF for 28 days; HLU: Rats were suspended, unloaded with −30° downward head tilting, and raised in a wooden box; HMF: Rats were raised normally in a GMF-shielded room; HMF+HLU: HLU rats were raised in a GMF-shielded room. Serum concentrations (mg L^−1^) of (A) iron, (B) calcium, (C) copper, (D) magnesium, (E) manganese, and (F) zinc. *P<0.05, **P<0.01.

The changes in the concentrations of trace elements in each group are shown in Figure 8. Two-way ANOVA showed that the interaction between the two factors (HLU*HMF) was statistically significant only in the serum iron concentrations.

The serum iron concentrations in the HMF and HMF+HLU groups were significantly greater than those in the BL, control and HLU groups (*P*<0.01); furthermore, the serum iron concentration was significantly higher in the HMF+HLU group than in the HMF group (*P*<0.01). However, the serum calcium concentrations were significantly lower in the HLU, HMF, and HMF+HLU groups than in the BL and control group (*P*<0.05, *P*<0.01, and *P*<0.01, respectively).

The serum copper concentrations tended to be greater in the HLU, HMF, and HMF+HLU groups than in the control group, although a significant difference was observed only between the HMF+HLU and control groups (*P*<0.05). The serum magnesium concentrations of the HMF and HMF+HLU groups were significantly lower than those of the BL, control and HLU groups (*P*<0.01). A high serum manganese concentration was noted in the control group but not in the other groups, which was lower level in the BL group, and was not detected in the HLU, HMF and HMF+HLU groups. All experimental groups had significantly lower serum zinc concentrations compared with the BL and control group (*P*<0.05, *P*<0.01, and *P*<0.01, respectively); the serum zinc concentrations were lower in the HMF and HMF+HLU groups and were significantly different between the HMF+HLU and HLU groups (*P*<0.05).

## Discussion

This land-based animal study used tail-suspended HLU rats to simulate weightlessness. Experimental animals were kept in an illumination- and ventilation-controlled GMF-shielding chamber to simulate the hypomagnetic and microgravity environment in deep space. Using tail-suspended HLU rats or mice as an animal model of simulated weightlessness to study bone loss in space is widely approved[29]. In the present study, rats were exposed to an HMF and simulated microgravity. The biomechanical properties, 2D BMD and BMC, were assessed, and a quantitative analysis of femoral trabecular and cortical bone using micro-CT was performed, to determine the effects of an HMF with and without simulated microgravity on the skeletal system. In addition, serum biochemical analysis and femur immunohistochemistry were performed to determine the possible mechanisms through which an HMF and simulated weightlessness influence the skeletal system.

Body weight has a close relationship with bone. Because the Sprague-Dawley rats we used were still in their growing period during the 28-day experiment, the weights of the rats in each group significantly increased. Nevertheless, the weight gain in the 2 tail-suspension groups was significantly less than that in the other 2 groups, possibly because unloading induces resistance to insulin-like growth factor-I[30] and transforming growth factor-β2[31]. There were no significant differences in weight gain between the HLU and HMF+HLU groups, implying that the HMF environment had no effect on body weight.

BMD and BMC rise in direct proportion to the rat's growth period. However, this increase was notably suppressed in the HLU and HLU+HMF groups, the latter of which shows particularly obvious effect. DEXA revealed that BMD and BMC were significantly lower in the HLU and HMF+HLU groups than in the control group; BMD was lower in the HMF+HLU group than in the HLU group. This indicates that HMF further promotes the reduction of femur BMD in HLU rats, whereas HMF alone does not cause a BMD reduction.

The biomechanical tests revealed that the ultimate loads of the HLU and HMF+HLU groups were significantly lower than that of the control group, whereas no significant differences were observed in these values between the HLU and HMF+HLU groups or the HMF and control groups. This indicates that the hypomagnetic environment in this study did not significantly influence the ultimate load of the femur in the 3-point bending test. Therefore, HLU is the primary reason for the decreased ultimate load. With regard to the toughness factor and elastic modulus, compared with the control and HMF groups, the femurs of the HMF+HLU and HLU groups exhibited higher toughness and lower elasticity, which led to reduced load tolerance capacity, thereby increasing the chance of bone fracture. Compared with the HLU group, the HMF+HLU groups exhibited decreased femur toughness and elasticity. Although BMD was significantly reduced in both groups, for growing rats, the mechanical properties differed, indicating that the HMF environment might have influenced the reconstruction characteristics of bone minerals inside the femur. This is the first study demonstrating that HMFs might affect the bone reconstruction process in vivo. The finding that a static magnetic field can affect bone reconstruction both in vivo and in vitro corroborates the importance of magnetic fields in bone reconstruction from another perspective[32]–[35].

To confirm the effect of HMF on bone reconstruction, micro-CT was used to further analyze changes in cancellous bone trabeculae. In the selected ROI of 3D cancellous bone, each group exhibited consistent trends regarding BMD, BMC, and TMC; these parameters were significantly lower in the HLU and HMF+HLU groups than in the BL, control and HMF groups, and the parameters were even lower in the HMF+HLU group than in the HLU group. However, no significant changes in TMD was observed between the experimental groups, but was higher than in the BL group. TMD is defined as the BMD of the area considered to be skeletal, according to the threshold binarization within an ROI, following exclusion of the nonskeletal parts. The lack of a significant difference in TMD indicates that bone reconstruction did not influence the physical appearance of bone tissue. BMD, BMC and TMC in HLU and HMF+HLU groups were lower than in the BL group, which declare that bone formation was inhibited, especially in HMF+HLU. HLU inhibits the process of bone formation in which HMF increases the inhibiting level. The extent of the decreases in the 3D BMD and BMC of cancellous bone within the ROI was greater than that in the 3D data obtained by DEXA, indicating that bone mineral loss in the HLU and HMF+HLU groups mainly involved cancellous bone. Bone loss was simultaneous with bone remodeling.

The shape of bone trabeculae can be described by the SMI[26]. In osteoporosis, bone trabeculae are converted from a plate shape to a rod shape, and the SMI is increased. The HLU and HMF+HLU groups had almost rod-shaped bone trabeculae, and both exhibited osteoporosis. The control and HMF groups had smaller SMI values and exhibited plate-shaped trabeculae. But, their significant differences were still existing, which implied that HMF affected the bone remodeling. However, the SMI is insufficient to describe the degree of osteoporosis. The BVF (or BV/TV), Euler number, and connectivity density (1/mm^3^) of bone trabeculae can be used to further determine the degree of osteoporosis[27], [28]. For growing rats, BVF in control and HMF groups were no obvious changes and the HMF environment alone does not cause the changes of BVF. But in the HLU and HMF+HLU groups, all of these parameters were significantly lower than in the BL, control and HMF groups and in the HMF+HLU group the values were the lowest. Hence, the microscopic 3D structure of femurs in the present study further confirms that HMF aggravates bone loss in HLU rats and that the HMF environment alone does not cause bone loss in rats.

The degree of anisotropy of bone trabeculae indicated certain changes in the directionality and symmetry of bone trabecular structure among the experimental groups; in particular, the changes in major/minor axis ratios of a1/a2 and a2/a3 in the oval planes were significantly different between the HMF+HLU and HLU groups. This indicates that the HMF environment influences the directionality and symmetry of the bone trabecular structure during reconstruction, which may affect the biomechanical properties of the femur, thus resulting in the observed decreases in femur toughness and elasticity in the HMF+HLU group.

Data regarding the x, y, and z axial directions as well as their mean values in cancellous bone ROI were obtained using MicroView™. The BV/TV value of normal growth rats was basically unchanged and the single factor of HMF environment could not affect it, while HLU and HMF+HLU caused a significant reduction of BV/TV. And simultaneously, a significant difference was observed in this value between the HMF+HLU and HLU groups. Moreover, BS/BV and Tb.Th exhibited consistent changes in all 3 axial directions, and no significant differences were observed in these values between the HMF+HLU and HLU groups. The inconsistencies with respect to BV/TV and BS/BV indicate that although osteoporosis was observed in the HMF+HLU and HLU groups, their bone trabeculae had different appearances. Even though the HMF+HLU and HLU groups showed no significant changes in Tb.Th, the former exhibited significantly decreased trabecular number and significantly increased Tb.Sp. These findings suggest increased bone resorption. Hence, we concluded that osteoporosis was more severe in the HMF+HLU group than in the HLU group.

In the case of bone loss, the loss of cortical bone always occurs after that of cancellous bone. In this study, BMC and mean cortical bone thickness was apparently decreased in both the HLU and HMF+HLU groups, showing that the HMF environment affected not only cancellous bone but also cortical bone; this decrease was particularly greater in the HMF+HLU group.

With regard to immunohistochemistry, because tissue section selection was inconsistent in each group, normalization was difficult. However, apparent RANKL expression was observed on the femoral bone trabecular surface in both the HMF+HLU and HLU groups. High RANKL expression is the major cause of bone loss. Osteoblasts on the bone trabecular surface form cell-cell contacts with preosteoclasts via RANKL expression on the cell surface to promote osteoclast formation[36]. Mature osteoclasts erode the bone trabecular surface and cause bone loss.

The serum concentrations of bALP, DPD and GC in the BL group were in higher level, which may be related to vigorous metabolism of young rats. As the growth of the age, theirs concentrations were significantly lower. But in the three challenge groups, theirs concentrations were maintained in higher level. The bALP concentration is a common index used to evaluate bone formation and turnover; bALP is secreted by osteoblasts and reflects osteoblast activity. It hydrolyzes phosphates during osteogenesis to provide the phosphoric acid required for hydroxyapatite deposition, while simultaneously hydrolyzing pyrophosphates and preventing their inhibition of bone mineral formation, thus promoting bone formation[37]. The ALP expression of unloaded skeleton is considered to be significantly reduced based on the finding of decreased proliferation ability of osteoblasts in tail-suspended rats[29]. The early serum ALP level of unloaded rats also shows a decreasing trend. However, after 2 weeks of unloading, the serum ALP level returns to normal[38]. In this study, the unloading of rats lasted for 28 days, and the serum ALP level increased significantly. This phenomenon may be related to feedback regulation caused by the early decrease in the serum ALP level. DPD is only present in type I collagenous fiber of bone and is released into the blood as the degradation product of type I collagenous fibers, when osteoclasts are active. DPD is unaffected by diet and can be used as a specific index to reflect bone resorption. Changes in DPD reflect the degree of bone resorption during the bone turnover process[39], [40]. In our study, serum bALP and DPD concentrations increased significantly to different extents in each experimental group, indicating active bone tissue metabolism in each group and significantly elevated bone formation and resorption.

Interestingly, the serum GC content was significantly increased in each experimental group. An excessive level of GC is the most common cause of osteoporosis. GCs can directly act on osteoblasts and osteocytes to induce apoptosis, thus reducing bone formation[41], [42]. Increases in GCs are usually caused by stress responses to changes in the surrounding environment. Excessive GC levels further promote the production of reactive oxygen species (ROS)[43], [44]. Under normal physiological conditions, ROS produced by osteoclasts stimulate and promote bone tissue resorption[45], [46]. Oxidative stress increases the sensitivity of osteocytes to GCs. GCs can directly act on osteoblasts, and reduce the number of osteoblasts via both oxidative stress-dependent and -independent pathways[47]. When ROS production exceeds the capacity of the natural antioxidant defense mechanisms of the body, related oxidative stress responses cause major bone loss and weaken bones, and may even manifest certain characteristics of osteoporosis[48]–[50]. This is because ROS such as H_2_O_2_ and superoxide anion can stimulate bone marrow mesenchymal cells and osteoblasts to express RANKL, consequently inducing osteoclast differentiation and maturation as well as promoting bone resorption[51]–[53].

Maintaining a normal biological rhythm is important to avoid excessive oxidative stress responses[54]. Changes in GMF activity must reach a certain magnitude in order to affect the amplitude of melatonin production. If the fluctuation is sufficiently large (i.e., >80 nT/3 h), it significantly affects melatonin concentration in saliva[55]. Because life on Earth has adapted to the existing GMF, the much weaker HMFs (i.e., <300 nT in the present study) are novel environments for organisms. In environments in which there are no circadian rhythm changes due to the GMF but only a constant extremely weak magnetic field, the biological rhythms of animals change[56], [57]. In a direct response to this change, the 24-hour circadian melatonin secretion rhythm is disturbed, and melatonin itself can weaken the effect of GCs[58], thus decreasing the body's antioxidant capacity[59]. In addition, to adapt to HMF environments, endogenous GC secretion by the adrenal glands is triggered via the pituitary-adrenal axis, to increase blood GC concentrations, thus resulting in more intense oxidative stress responses[60].

Long-term GMF deprivation alters the concentrations of trace elements in rats[61]. A perturbed balance of trace elements in the body affects metabolic processes. Trace elements are closely associated with the development of osteoporosis[62]; sufficient zinc, copper, magnesium, calcium, and manganese uptake is critical for maintaining a healthy skeleton. In the present study, the serum iron concentrations of rats in the HMF and HMF+HLU groups were significantly higher (approximately double) than those of the BL, control and HLU groups, indicating that HMF environments cause iron accumulation in the body. Serum calcium, magnesium, and zinc concentrations in each experimental group were decreased to different degrees. Magnesium specifically decreased in the 2 groups of rats subjected to the HMF environment, indicating that the pituitary adrenal axis affects the blood magnesium concentration. Although we used graphite furnace atomic absorption spectroscopy, which is high sensitivity for detecting trace elements, serum manganese was not detected in any experimental group.

Iron is a trace element that has important functions in vivo[63]. In the skeletal system, both excess and insufficient iron can reduce bone mass[64]–[66]. In vitro, iron can even inhibit the growth of hydroxyapatite crystals[67]. Iron ions promote RANKL-induced osteoclast differentiation, accompanied by increased ROS levels and oxidative stress responses. Excess ROS production confirms that iron ions promote osteoblast differentiation and bone resorption[68], [69]. A study on the relationships between the serum iron levels, oxidative stress, and bone resorption of astronauts living for a long duration on the International Space Station revealed that variations in serum iron concentration are positively correlated with an oxidative stress marker, 8-hydroxy-2′-deoxyguanosine, suggesting that increased iron storage during spaceflight might be a risk factor that causes oxidative damage and bone resorption[70].

Zinc is an important enzyme reaction catalytic factor and structural cofactor for many enzymes and some other proteins. Although Zn^2+^ has no redox activity under physiological conditions, numerous studies demonstrate that zinc deficiency intensifies the oxidative stress responses, resulting in oxidative damage to DNA, proteins, and lipids. Thus, zinc has an indirect antioxidant effect[71]. Moreover, zinc deficiency causes DNA damage by increasing oxidative stress and blocking DNA repair[72]. Thus, zinc plays an important role in maintaining DNA integrity. Zinc deficiency in growing rats reduces bone mass accumulation, which might be an important pathogenic mechanism of osteoporosis[73]. Insulin treatment has considerable efficacy against diabetic osteoporosis. However, the efficacy of this treatment is greatly reduced in diabetic osteoporosis combined with zinc deficiency[74].

Manganese deficiency can cause disorders of serum bone-regulating hormones and bone metabolic enzymes[75]. Moreover, manganese supplementation can effectively inhibit bone loss in ovariectomized rats[76].

Decreases in serum calcium concentration can directly cause parathyroid hormone (PTH) secretion, thus triggering bone calcium release. One of the underlying mechanisms for this action involves the effect of PTH on osteocytes; PTH promotes cytoplasmic processes to release alkaline phosphatase and proteolytic enzymes into the surrounding environment, thus accelerating dissolution of the walls of bone canaliculi. Another mechanism involves the increase in the stimulation of osteoblasts to express RANKL, thus inducing the differentiation and maturation of preosteoclasts, which then increase the number of osteoclasts and accelerate bone dissolution and resorption. Therefore, continuous low serum calcium levels increase the risk of osteoporosis[77].

Both high and low magnesium levels adversely affect the skeleton; therefore, strictly controlling magnesium balance is vital to bone health[78]. The most direct effect of magnesium deficiency on the skeletal system is the change in the hydroxyapatite crystal structure of bone tissue through its effect on osteocytes, consequently weakening the load-bearing capacity of bones[79]. Magnesium deficiency is related to decreased PTH and vitamin D levels. Moreover, magnesium supplementation can correct osteoporosis combined with PTH and vitamin D deficiency[80]. Magnesium deficiency is related to low-grade inflammation, and the inflammatory cytokines produced by inflammatory responses stimulate bone reconstruction and decrease bone mass[81]. In addition, magnesium deficiency causes endothelial dysfunction, and the resultant vascular disease is also a risk factor for osteoporosis[82], [83].

In this study, femoral BMD decreased significantly in the HLU group, demonstrating that HLU is a reliable model for studying weightlessness-induced bone loss in rats. The additional decrease in femoral BMD in the HMF+HLU group indicates that HMF environments further promote bone loss in HLU rats. However, in the HMF group, which was conventionally housed in a hypomagnetic environment, although serum GC, DPD, and bALP concentrations increased and trace elements were adversely altered, the femurs did not exhibit bone loss. One of the reasons for this is that the skeletal systems of HMF rats were continuously stimulated by gravity, which is the most important environmental factor that maintains bone health[84]. Another possible reason is that even though the HMF environment caused oxidative stress responses in HMF rats, these responses did not exceed the rats' antioxidant capacity. Moreover, in HLU rats, bone loss directly caused by hindlimb unloading, the −30° downward head-tilting position also caused a stress responses in rats simultaneously, which, combined with the effect of the HMF environment, increased the oxidative stress responses in the body, resulting in increased bone loss. This interesting phenomenon requires more direct evidence for confirmation in future studies.

In conclusion, HMFs can promote additional bone loss in rats in simulated weightlessness. The underlying mechanism might involve changes in biological rhythms by the HMF, which may induce oxidative stress responses; in turn, excessive levels of ROS stimulate osteoblasts to secret RANKL and promote the maturation and activation of osteoclasts, eventually causing bone resorption. In addition, changes in the concentrations of trace elements in the rats' bodies caused by HMFs might also be one of the important reasons for the oxidative stress responses.
